# From attachment anxiety to short video addiction: the roles of attentional control and alexithymia

**DOI:** 10.3389/fpsyg.2026.1764536

**Published:** 2026-03-26

**Authors:** Haodong Su, Dan Luo, Hongyu Wang, Xiaodong Li, Ye He

**Affiliations:** 1 College of Humanities, Anhui Science and Technology University, Chuzhou, China; 2 Department of Psychiatry of Women and Children, The Second People's Hospital of Guizhou Province, Guiyang, China; 3 Department of Geriatric Psychiatry, The Second People's Hospital of Guizhou Province, Guiyang, China

**Keywords:** alexithymia, attachment anxiety, attentional control, sequential mediation, short video addiction

## Abstract

**Introduction:**

Short video addiction is a prevalent behavioral phenomenon among young adults, but its underlying mechanisms are not well understood. Guided by attachment theory, this study examined associations between attachment anxiety, attentional control, alexithymia, and short video addiction.

**Methods:**

A total of 342 Chinese university students completed self-report measures assessing attachment anxiety, attentional control, alexithymia, and short video addiction. Mediation analyses were conducted to investigate the indirect associations between attachment anxiety and addictive tendencies through attentional control and alexithymia.

**Results:**

Attachment anxiety was positively associated with higher levels of short video addiction. Indirect associations were observed via two pathways: lower attentional control and higher alexithymia. Additionally, a sequential association was identified, whereby lower attentional control was linked to higher alexithymia, which in turn was related to stronger addictive tendencies.

**Discussion:**

These findings provide an integrative framework describing how attachment-related vulnerabilities relate to cognitive and emotional characteristics associated with short video addiction. The results highlight potential factors to consider in strategies aimed at preventing or addressing addictive behaviors among young adults.

## Introduction

### Short video addiction

With the rapid development of mobile internet technologies, short-form video applications such as TikTok and Kuaishou have become deeply integrated into contemporary life, functioning as a primary channel for entertainment and information consumption (Li et al., 2025). By 2023, the number of short video users in China had exceeded one billion, with average daily usage time continuing to increase (Lu et al., 2022). Owing to their brevity, high informational density, algorithm-driven personalization, and instant feedback, short videos efficiently stimulate the brain's reward circuitry, eliciting dopamine release and generating transient states of pleasure (Tian et al., 2023). Nevertheless, this mode of fragmented information processing and immediate gratification renders users highly susceptible to dysregulated engagement, thereby fostering a newly recognized form of behavioral addiction—short video addiction (Chen et al., 2022).

Short-form video addiction (SVA) is conceptualized as a pattern of excessive, maladaptive, or highly dependent engagement with short-form video applications, characterized by difficulties in regulating usage, tendencies to use videos for coping or escape, and reduced efficiency in daily activities (Liao, 2024). This definition emphasizes behavioral tendencies rather than clinical-level addiction. The problem is particularly salient among young populations such as university students, with prevalence rates reported as high as 27.12% (Yuxue et al., 2023). Growing empirical evidence suggests that short video addiction poses significant threats to both psychological and physical health, as well as to everyday functioning. At the cognitive level, prolonged immersion in fragmented streams of short video content undermines information processing, leading to impairments in attentional control, executive functioning (particularly inhibitory control), and working memory, and may even precipitate attentional deficits (Chen et al., 2022). At the level of mental health, short video addiction has been consistently linked to depression, anxiety, emotional numbing, and elevated stress, with long-term consequences that may alter neural functioning and diminish overall wellbeing (Ye et al., 2022). At the level of social functioning, these cognitive and affective impairments ultimately manifest in tangible life difficulties, such as declines in academic performance (Xie et al., 2023).

Importantly, short-form video addiction should be understood within its broader sociocultural context. Patterns of digital media use, norms surrounding emotional expression, and socially sanctioned coping strategies vary across cultural settings and may shape how individuals engage with short-form video platforms (Khoury and Hemsley, 2025; Ning, 2025; Xiong et al., 2024). In cultural contexts where emotional restraint, interpersonal harmony, or indirect emotion regulation are emphasized, individuals may be more inclined to rely on external, technology-mediated activities as accessible means of managing internal distress or attentional disengagement (Chen et al., 2025; Pruessner and Altan-Atalay, 2025). Within such contexts, short-form video use may become particularly salient as a routine, socially acceptable form of emotional distraction and self-regulation (Chen, 2025; Nguyen et al., 2025). Accordingly, examining psychological correlates of short-form video addiction within a specific cultural setting allows for a more context-sensitive understanding of individual differences in vulnerability, without presupposing causal relationships between culture and addictive behaviors.

### Attachment-based perspective on short-form video addiction

While existing research has primarily emphasized the technological affordances of short-form video platforms—such as algorithmic recommendation and instant feedback—in accounting for short-form video addiction, such perspectives remain insufficient to explain substantial individual differences in vulnerability to problematic use (Liu et al., 2025). From a developmental psychopathology perspective, susceptibility to behavioral addiction is not solely associated with external technological features, but is closely linked to relatively stable self-regulatory patterns shaped through early interpersonal experiences (Brand et al., 2025).

Attachment theory offers a comprehensive developmental framework for understanding these individual differences. According to attachment theory, early interactions with primary caregivers are internalized as internal working models of the self and others, which are associated with long-term patterns of emotion regulation, cognitive control, and coping strategies across the lifespan (Eichenberg et al., 2024; Huang et al., 2025). Importantly, insecure attachment—particularly attachment anxiety—has been consistently associated with difficulties in the regulation of attention and emotion (Messina et al., 2023), two psychological processes that have been repeatedly implicated in various forms of behavioral addiction (Liang et al., 2021; Park and Lee, 2022).

From this perspective, short-form video addiction may be conceptualized as a compensatory behavioral tendency linked to attachment-related vulnerabilities in self-regulation. Individuals with higher levels of attachment anxiety are more likely to engage in short-form video use as a readily accessible means of managing distress, seeking external validation, or diverting attention away from aversive internal experiences, rather than as a direct consequence of attachment insecurity. This attachment-based perspective thus provides an overarching theoretical structure that integrates distal developmental characteristics with proximal cognitive and emotional correlates, offering a coherent conceptual foundation for the mediation and serial mediation models examined in the present study.

### Attachment anxiety and short video addiction

As mentioned above, Attachment theory provides an important developmental framework for understanding individual differences in vulnerability to behavioral addictions. The theory posits that the close emotional bonds formed with primary caregivers early in life become internalized as “internal working models” of self–other relationships, which subsequently shape emotional regulation, cognitive processing, and interpersonal behavior across the lifespan (Dagan et al., 2021). Insecure attachment patterns, particularly attachment anxiety, have been identified as significant risk factors for a range of psychopathologies (Zhang et al., 2022). Individuals with attachment anxiety are characterized by an intense fear of rejection and abandonment, negative self-evaluations, and a tendency to excessively seek reassurance and closeness from others in order to mitigate inner insecurity (Coan and Sbarra, 2015).

A substantial body of evidence has demonstrated robust positive associations between attachment anxiety and various forms of online addictive behaviors, including social media addiction (Young et al., 2020). The underlying mechanism has often been explained through the lens of “compensatory” internet use: for individuals with high attachment anxiety, offline social interactions are fraught with uncertainty and the risk of rejection, whereas short video platforms and other social media provide a relatively safe and controllable virtual environment (Gregorini et al., 2025). Within this space, they can publish content, receive “likes” and comments, and thereby obtain external validation to fulfill their strong need for attention and approval, temporarily alleviating their internal anxiety and insecurity (Chen, 2019). However, this compensatory use of short videos as a means of regulating negative affect can readily escalate into uncontrolled, addictive patterns of behavior.

### Attachment anxiety and attentional control

Attachment anxiety not only shapes individuals' interpersonal strategies but also profoundly influences underlying cognitive processing, particularly attentional control (Chen et al., 2022). Attentional control refers to the ability to deliberately suppress irrelevant information and flexibly shift attention toward goal-directed behaviors when facing distractions or conflicts, representing a core component of executive functioning (Begum, 2025). According to Attentional Control Theory, anxiety impairs the functioning of top-down, goal-directed attentional systems while simultaneously enhancing the influence of bottom-up, stimulus-driven attention (Eysenck et al., 2023). Attachment anxiety can be conceptualized as a chronic, relationship-focused form of anxiety. Individuals with high attachment anxiety are in a state of persistent hypervigilance toward potential cues of rejection, with their limited attentional resources automatically and continuously allocated to monitoring threat-related information (Wauthia and Rossignol, 2016). Such sustained worry and rumination, known as hyperactivating strategies, consume substantial working memory resources, thereby directly undermining inhibitory and shifting functions that underlie attentional control (Bruning et al., 2023). In other words, the ongoing sense of interpersonal threat associated with attachment anxiety places the cognitive system in a prolonged stress mode, limiting the individual's ability to disengage attention from social threat concerns and reallocate it to current tasks, resulting in observable deficits in attentional control.

### Attachment anxiety and alexithymia

Alexithymia is not characterized by the absence of emotions but rather by deficits in the cognitive processing and regulation of emotional experiences (Lesser, 1981). Its core features include difficulty identifying one's own feelings, difficulty describing feelings to others, and an externally oriented thinking style, in which individuals focus on external details while neglecting internal emotional experiences (Hogeveen and Grafman, 2021). From a developmental perspective, the emergence of alexithymia is closely linked to early attachment experiences. Attachment anxiety often originates from inconsistent, unreliable, or intrusive responses by caregivers to a child's emotional needs (Kinnaird et al., 2019). In such environments, the child's emotional expressions may go unacknowledged, be misinterpreted, or even be punished. Over time, the child fails to learn how to identify, understand, and label complex internal feelings, resulting in a blurred and disorganized emotional world that ultimately manifests as the characteristic features of alexithymia (Farina and Schimmenti, 2025). These findings indicate a close association between attachment anxiety and alexithymia, with insecure attachment serving as a significant predictor of alexithymic traits.

### Attentional control and short video addiction

Deficits in executive functions, such as attentional control, are widely recognized as core vulnerability factors for behavioral addictions (Ocasio, 2011). Individuals with weaker attentional control exhibit impaired top-down cognitive regulation, making it difficult to inhibit dominant responses triggered by external temptations (e.g., smartphone notifications) or internal impulses (e.g., the urge to watch videos) (Yan et al., 2024; Ye et al., 2025). Once engaged in short video viewing, they struggle to disengage voluntarily from this highly stimulating, high-reward activity and to redirect their attention toward tasks requiring greater cognitive effort but yielding delayed rewards, such as studying or work (Zhu and Fong, 2025). Moreover, the maintenance of addictive behaviors is closely associated with attentional bias, whereby the attentional system is automatically and preferentially captured by cues related to the addictive behavior (Su et al., 2024). In the context of short videos, algorithmically driven streams of endless, novel, and engaging content create highly salient distractions for individuals with limited attentional control, effectively “locking” their attention onto the application interface (Al-Leimon et al., 2025). Importantly, the fragmented and fast-paced nature of short video content further impairs attentional control, creating a vicious cycle in which attentional deficits contribute to addiction, which in turn exacerbates attentional deficits (Cox et al., 2014).

### Alexithymia and short video addiction

According to the self-medication hypothesis, addictive behaviors often function as maladaptive strategies for coping with negative emotions (Utkarsh and Kanwar, 2025). Individuals with alexithymia, due to deficits in effectively identifying, understanding, and regulating emotions, experience internal affective states that are often confused, undifferentiated, and distressing in nature. Consequently, when confronted with stress or negative affect, they are unable to manage these internal states through adaptive strategies such as introspection, emotional expression, or seeking social support (Luo et al., 2022). In this context, short videos present a highly appealing “solution.” The immersive and highly stimulating content can effectively redirect attention away from a confusing internal world toward a rich and engaging external environment, serving as a form of cognitive and emotional escape (Gundogmus et al., 2021). By engaging with short videos, individuals with alexithymia can temporarily alleviate unbearable inner emptiness and discomfort, achieving immediate emotional relief. This emotion-avoidance–driven pattern of behavior constitutes a critical pathway to the development of addiction (Orsolini, 2020).

In summary, attachment anxiety, as a deep-seated personality trait rooted in early developmental experiences, may serve as a distal risk factor for the propensity to develop short video addiction. However, this influence is unlikely to be direct. A model more consistent with developmental psychopathology posits that attachment anxiety increases vulnerability to addictive behaviors by undermining individuals' core self-regulatory capacities. These core self-regulatory capacities include cognitive mechanisms, such as attentional control, and emotional mechanisms, encompassing emotional awareness and regulation, the converse of alexithymia. Importantly, there is currently a lack of integrative models that systematically examine how these factors interact to contribute to short video addiction. In particular, the mediating roles of attentional control and alexithymia in the relationship between attachment anxiety and short video addiction, as well as the potential interrelation between these two mediators, remain insufficiently empirically tested.

**Figure 1:**
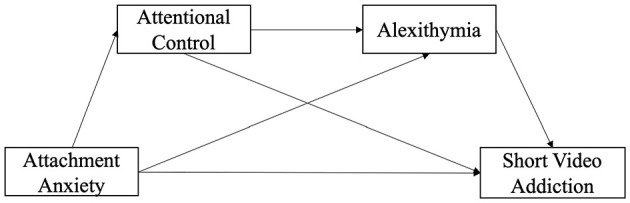
The serial mediation model hypothesized in the present study.

Building on the above, the present study aims to construct and examine a serial mediation model to explore the potential pathways through which attachment anxiety is associated with short-form video addiction via attentional control and alexithymia. Specifically, the study proposes the following hypotheses: Hypothesis 1: attachment anxiety is related to short-form video addiction through attentional control (AA → AC → SVA); Hypothesis 2: attachment anxiety is related to short-form video addiction through alexithymia (AA → Ale → SVA); Hypothesis 3: attachment anxiety is associated with short-form video addiction via a sequential pathway involving attentional control and alexithymia (AA → AC → Ale → SVA). The hypothesized model proposed in this study is shown in Figure 1.

The theoretical contribution of this study lies in integrating distal attachment factors with proximal cognitive (attentional control) and emotional (alexithymia) variables, providing a more nuanced framework for understanding the development of short-form video addiction. From a practical perspective, identifying potential associations and pathways may help inform targeted strategies for preventing or mitigating problematic short-form video use. Overall, this study seeks to provide empirical insights into factors associated with short-form video addiction and to contribute to the literature on digital behavioral outcomes.

## Materials and methods

### Participants and procedures

Data for the present study were collected in December 2024. A total of 364 university students were recruited from a comprehensive university in China. Participants completed the survey on the https://www.wjx.cn/platform under the guidance of the experimenter in classroom settings. Upon completing the survey, participants received a small gift (either a keychain or a decorative bookmark), with the exact value of the compensation disclosed only after survey completion. Participation was entirely voluntary, and all participants provided written informed consent prior to completing the questionnaire.

To ensure data quality, several exclusion criteria were applied: (1) participants who provided identical responses to half or more of the items on any scale (Curran, 2016); (2) participants whose total survey completion time fell outside the 10–20 min range; and (3) participants who were not undergraduates. After applying these criteria, the final sample consisted of 342 undergraduate students aged 18–22 years (246 males and 96 females; demographic details are presented in Table 1). The study protocol was approved by the Ethics Committee of Anhui Science and Technology University.

**Table 1:** Descriptive statistics.

**Variable**	***N***	**(%)**	**Variable**	***M***	**SD**
**Gender**					
Male	246	71.93	Age	19.98	1.08
Female	96	28.07			
**Grade**					
Freshman	312	91.23			
Sophomore	30	8.77			

Male = 1; Female = 2; Freshman = 1; Sophomore = 2.

### Measures

#### Attachment anxiety

Attachment anxiety was assessed using the Chinese adaptation of the Attachment anxiety Scale in 2021 (e.g., “I feel distressed when important others do not understand me”). This scale was modified from the Experiences in Close Relationships (ECR) questionnaire (Brennan et al., 1998). In the adaptation, the “attachment security” dimension, which demonstrated poor reliability and validity, was removed, retaining only the “attachment anxiety” dimension. This makes the scale more suitable for measuring general attachment anxiety, rather than being restricted to romantic partners (Zhang, 2021). The attachment anxiety scale comprises eight items, rated on a 5-point Likert scale (1 = strongly disagree, 2 = disagree, 3 = uncertain, 4 = agree, 5 = strongly agree), with all items positively keyed. The total score across all items served as an indicator of attachment anxiety, with higher scores reflecting higher levels of attachment anxiety. In the present study, the scale demonstrated excellent internal consistency, with a Cronbach's alpha of 0.900 and a McDonald's ω of 0.903.

#### Attentional control

Attentional control was assessed using the Attentional Control Scale (ACS) (Derryberry and Reed, 2002), which evaluates individuals' capacity to regulate attention (e.g., “In a noisy environment, I find it difficult to concentrate on a demanding task”). The ACS consists of 20 items and includes two subscales: attentional focusing (items 1–9) and attentional shifting (items 10–20). Items are rated on a 4-point Likert scale (1 = strongly disagree, 2 = disagree, 3 = agree, 4 = strongly agree), with 11 items reverse-scored. The total score across all items served as an index of attentional control, with higher scores indicating better attentional control ability. The ACS has been widely applied in China and demonstrates good reliability and validity (Yu, 2020). In the present study, the scale exhibited satisfactory internal consistency, with a Cronbach's alpha of 0.842 and a McDonald's ω of 0.864.

#### Alexithymia

Alexithymia was assessed using the Chinese revised version of the Toronto Alexithymia Scale (TAS-20) (Ling et al., 2009), which measures individuals' difficulties in identifying and describing emotions (e.g., “I often find it hard to pinpoint my inner feelings”). The scale comprises 20 items rated on a 5-point Likert scale (1 = strongly disagree, 2 = disagree, 3 = neutral, 4 = agree, 5 = strongly agree). The mean score across all items was calculated to index the level of alexithymia, with higher scores indicating greater difficulties in emotional awareness and processing. In the present study, the TAS-20 demonstrated good internal consistency, with a Cronbach's alpha of 0.822 and a McDonald's ω of 0.860.

#### Short video addiction

SVA was assessed using the Short-Form Video Addiction Scale (SFVAS), developed for use with Chinese university students (Xie et al., 2023). The SFVAS adapts the Internet Addiction Diagnostic Questionnaire (IADQ) (Young, 1998), with two modifications: (1) replacing “Internet/online” with “short-form video,” and (2) using a 5-point Likert scale ranging from 1 (almost never) to 5 (always). Items include statements such as “I always spend more time on short-form video applications than I originally planned.” The SFVAS comprises 20 items, and the mean score was used to index the degree of behavioral tendencies toward short-form video overuse. Higher scores reflect greater tendencies rather than clinical addiction. In this study, the scale demonstrated excellent internal consistency (Cronbach's α = 0.921 and McDonald's ω = 0.924).

### Statistical analysis

Descriptive statistics and Pearson correlation analyses were conducted for all study variables using SPSS version 26.0. Following Hayes' recommendations, mediation analyses were performed using the PROCESS macro (v3.4). To clarify the internal structure of the proposed mechanism, mediation analyses were conducted in a stepwise manner. First, PROCESS Model 4 was employed to test the independent mediating roles of attentional control and alexithymia in the association between attachment anxiety and short-form video addiction. These analyses were intended to examine whether each variable functioned as a theoretically meaningful mediator representing distinct cognitive and emotional regulatory processes. Building on these results, PROCESS Model 6 was subsequently applied to assess a sequential mediation pathway, in which attentional control and alexithymia were specified as ordered mediators linking attachment anxiety to short-form video addiction. This serial mediation model was designed to integrate the two single mediation pathways into a theoretically grounded sequential process, rather than to provide an alternative explanation.

## Results

### Testing for common method bias

To address potential common method bias, Harman's single-factor test was conducted by including all items from the four scales in an exploratory factor analysis. The results indicated that the first factor accounted for 20.47% of the total variance, which is below the critical threshold of 40% (Podsakoff et al., 2003). These findings suggest that the likelihood of significant common method bias confounding the data interpretation is low.

### Descriptive statistics and correlation analyses

Table 2 presents the descriptive statistics and correlation coefficients among the study variables. Attachment anxiety was positively associated with short-form video addiction (*r* = 0.21, *p* < 0.01) and alexithymia (*r* = 0.39, *p* < 0.01), suggesting that higher attachment anxiety tends to co-occur with greater alexithymic traits and more severe short-form video engagement. Attentional control was negatively related to attachment anxiety (*r* = −0.19, *p* < 0.01), alexithymia (*r* = −0.21, *p* < 0.01), and short-form video addiction (*r* = −0.31, *p* < 0.01). Moreover, alexithymia showed a positive correlation with short-form video addiction (*r* = 0.46, *p* < 0.01).

**Table 2:** Descriptive statistics and correlation analyses of variables.

**Variable**	***M***	**SD**	**AA**	**AC**	**Ale**	**SVA**	**Skewness**	**Kurtosis**
AA	27.98	6.22	1				0.062	0.160
AC	49.78	5.61	−0.193^**^	1			0.587	3.040
Ale	3.10	0.53	0.385^**^	−0.208^**^	1		0.433	3.650
SVA	2.58	0.70	0.209^**^	−0.314^**^	0.455^**^	1	0.660	1.110

N = 342.

AA, attachment anxiety; AC, attentional control; Ale, alexithymia; SVA, short video addiction.

^**^p < 0.01.

Regarding the distribution of the variables, skewness values were 0.062 for attachment anxiety, 0.433 for alexithymia, 0.587 for attentional control, and 0.660 for short-form video addiction, indicating only mild asymmetry. Corresponding kurtosis values were 0.160 for attachment anxiety, 3.65 for alexithymia, 3.04 for attentional control, and 1.11 for short-form video addiction, suggesting slight deviations from normality. Although Shapiro–Wilk tests were statistically significant for all variables (*p* < 0.001), such results are common in large samples (*N* = 342). Overall, these descriptive statistics indicate that the distributions can be considered approximately normal, supporting the appropriateness of using parametric analyses, including correlation and mediation analyses, in the present study.

### Testing for mediation effect of attentional control and alexithymia

Previous research has shown that during the university years, Chinese students experience rapid social and psychological maturation due to reduced supervision from both family and the university environment (Geng et al., 2018). Consequently, students at different grade levels may exhibit differences in psychological and behavioral characteristics. Therefore, we included gender and grade as covariates in our data analyses, as these factors may influence the variables of interest in the current study. Independent-samples *t*-tests or ANOVAs were conducted to examine potential group differences. Results indicated significant gender differences in attachment anxiety (*t* = −2.86, *p* < 0.01) and attentional control (*t* = 3.38, *p* < 0.01). Although no significant differences were observed across grade levels for the variables of interest, gender and grade were nevertheless included as covariates in the subsequent model analyses to ensure rigor.

We tested Hypothesis 1 using Model 4 of PROCESS 3.4 in SPSS. The model output is presented in Table 3. As shown in Model 1, gender and attachment anxiety emerged as significant predictors of attentional control, with attachment anxiety negatively predicting attentional control. Furthermore, as indicated in Model 2, gender and attentional control significantly negatively predicted short-form video addiction, whereas attachment anxiety significantly positively predicted short-form video addiction. Results indicated that attentional control was negatively associated with short-form video addiction (β = −0.31). After accounting for attentional control, attachment anxiety remained positively associated with short-form video addiction (direct effect: β = 0.17). Importantly, the indirect effect of attachment anxiety on short-form video addiction via attentional control was statistically significant (indirect effect: β = 0.05), as the 95% confidence interval did not include zero, supporting Hypothesis 1.

**Table 3:** Regression coefficients, standard errors, and model summary information for the mediation effect of attentional control on short video addiction.

**Variable**	**Model 1 (AC)**				**Model 2 (SVA)**			
	**β**	***t***	**SE**	**95%CL**	**β**	***t***	**SE**	**95%CL**
Constant	56.43	32.15	1.76	52.97–59.88	4.29	10.18	0.42	3.46–5.12
Grade	0.01	0.11	0.96	−1.78 to 1.99	−0.02	−0.47	0.11	−0.28 to 0.17
Gender	−0.16	−2.85^**^	0.69	−3.33 to −0.61	−0.14	−2.65^**^	0.08	−0.39 to −0.06
AA	−0.17	−3.13^**^	0.05	−0.25 to −0.06	0.17	3.31^**^	0.01	0.01–0.03
AC					−0.31	−5.92^***^	0.01	−0.05 to −0.03
*R^2^*	0.06				0.14			
*F*	7.37				14.11			

AA, attachment anxiety; AC, attentional control; SVA, short video addiction.

^**^p < 0.01; ^***^p < 0.001.

Similarly, Hypothesis 2 was examined, with detailed model outputs presented in Table 4. Results indicated that gender significantly negatively predicted alexithymia in Model 1, while attachment anxiety significantly positively predicted alexithymia. Model 2 results indicated that the indirect association between attachment anxiety and short-form video addiction via alexithymia was statistically significant. Attachment anxiety was positively associated with alexithymia (β = 0.40), and alexithymia was positively associated with short-form video addiction (β = 0.43). The resulting indirect effect was β = 0.17, and the bootstrap 95% confidence interval did not include zero, supporting Hypothesis 2.

**Table 4:** Regression coefficients, standard errors, and model summary information for the mediation effect of alexithymia on short video addiction.

**Variable**	**Model 1 (Ale)**				**Model 2 (SVA)**			
	**β**	***t***	**SE**	**95%CL**	**β**	***t***	**SE**	**95%CL**
Constant	2.32	14.67	0.16	2.01–2.63	0.81	3.15	0.26	0.30–1.32
Grade	−0.01	−0.24	0.09	−0.19 to 0.15	−0.02	−0.42	0.11	−0.26 to 0.17
Gender	−0.11	−2.02^*^	0.06	−0.25 to −0.00	−0.05	−0.93	0.08	−0.23 to 0.08
AA	0.40	7.93^***^	0.00	0.03–0.04	0.05	0.93	0.01	−0.01 to 0.02
Ale					0.43	8.21^***^	0.07	0.43–0.70
*R^2^*	0.16				0.21			
*F*	21.44				22.66			

AA, attachment anxiety; Ale, alexithymia; SVA, short video addiction.

^*^p < 0.05; ^***^p < 0.001.

### Testing for serial mediation effect

**Figure 2:**
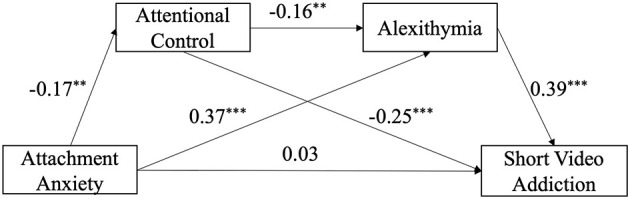
Theoretical research model with standard coefficients. Regression coefficients were obtained with grade and gender in covariates in PROCESS Procedure for SPSS (**p < 0.01; ***p < 0.001, *N* = 342).

Gender and grade were included as covariates in the analysis. Model 6 of PROCESS 3.4 was employed to examine the mediating roles of attentional control and alexithymia in the relationship between attachment anxiety and short-form video addiction (for detailed model outputs, see Table 5). Results indicated that, in Model 3, attentional control significantly negatively predicted short-form video addiction, whereas alexithymia significantly positively predicted short-form video addiction, supporting Hypothesis 3. The detailed path coefficients of the model are illustrated in Figure 2.

**Table 5:** Regression coefficients, standard errors, and model summary information for the influence of attachment anxiety in a model of short video addiction.

**Variable**	**Model 1 (AC)**			**Model 2 (Ale)**			**Model 3 (SVA)**		
	**β**	***t***	***SE***	**β**	***t***	***SE***	**β**	***t***	***SE***
Constant	56.43	32.15	1.75	3.18	10.11	0.31	2.67	5.99	0.45
Grade	0.01	0.11	0.96	−0.01	−0.23	0.09	−0.02	−0.41	0.11
Gender	−0.16	−2.85^**^	0.69	−0.13	−2.51^*^	0.06	−0.09	−1.81	0.08
AA	−0.17	−3.13^**^	0.05	0.37	7.39^***^	0.00	0.03	0.51	0.01
AC				−0.16	−3.15^**^	0.00	−0.25	−5.03^***^	0.01
Ale							0.39	7.53^***^	0.07
*R^2^*	0.06			0.18			0.27		
*F*	7.37			18.98			24.49		

AA, attachment anxiety; AC, attentional control; Ale, alexithymia; SVA, short video addiction.

^*^p < 0.05; ^**^p < 0.01; ^***^p < 0.001.

Additionally, we conducted a bias-corrected percentile bootstrap procedure with 5,000 resamples and 95% confidence intervals to test the chain mediation effects. An effect was considered statistically significant if the confidence interval did not include zero; results are presented in Table 6. The total effect of attachment anxiety on short-form video addiction was significant, whereas the direct effect of attachment anxiety → short-form video addiction was not significant, as its 95% confidence interval included zero. Regarding the indirect paths, Path 1 (attachment anxiety → attentional control → short-form video addiction (95% CI [0.0008, 0.0090]), Path 2 (attachment anxiety → alexithymia → short-form video addiction (95% CI [0.0088, 0.0262]), and Path 3 (attachment anxiety → attentional control → alexithymia → short-form video addiction (95% CI [0.0002, 0.0025]) were all significant, as their 95% confidence intervals did not include zero.

**Table 6:** Direct and indirect effects of attachment anxiety on short video addiction.

**Path way**	**Estimate**	**SE**	**95%CI**
Total effect	0.0251	0.006	0.0133–0.0370
Direct effect	0.003	0.0058	−0.0085 to 0.0144
Total indirect effect	0.0222	0.0052	0.0128–0.0332
AA → AC → SVA	0.0046	0.0021	0.0008–0.0090
AA → Ale → SVA	0.0164	0.0044	0.0088–0.0262
AA → AC → Ale → SVA	0.0012	0.0006	0.0002–0.0025

## Discussion

To clarify the respective roles of the single and serial mediation analyses within the overall analytical framework, it is important to note that the single mediation models were not intended as alternative explanatory accounts, but rather as analytically preparatory steps for the subsequent serial mediation model. Specifically, the two single mediation pathways—attachment anxiety (AA) → attentional control (AC) → short video addiction (SVA) and attachment anxiety (AA) → alexithymia (Ale) → short video addiction (SVA)—were examined to establish whether each mediator independently showed a statistically meaningful indirect association with AA and SVA. By demonstrating that both AC and Ale were individually positioned as viable intervening variables in the association between AA and SVA, these analyses provided an empirical basis for integrating the two mediators into a unified serial mediation structure. On this basis, the serial mediation model (AA → AC → Ale → SVA) was specified to assess whether the indirect associations observed in the single mediation analyses could be coherently represented within a sequential configuration that aligns with the conceptual ordering of the variables. Thus, the single mediation analyses functioned as foundational components that informed the specification and interpretation of the serial mediation pathway, rather than as competing interpretations of the AA–SVA relationship.

As one component of the overall mediation framework, the present study confirmed the mediating role of attentional control in the relationship between attachment anxiety and short-form video addiction (AA → AC → SVA), and the results showed that attachment anxiety was negatively associated with attentional control. According to Attentional Control Theory, anxiety is related to differences in top-down vs. bottom-up attentional processes (Begum, 2025). Individuals with high attachment anxiety may tend to show higher vigilance toward potential interpersonal rejection cues, showing a pattern of increased attention to social cues. Such persistent worry and rumination are related to lower working memory efficiency, thereby are associated with weaker attentional control that underlie attentional control (Bruning et al., 2023). In other words, the chronic anxiety associated with insecure attachment makes it difficult for individuals to effectively suppress irrelevant information—particularly internally generated thoughts related to interpersonal threat—resulting in a generalized reduction in attentional control.

Complementing the cognitive pathway, the present findings also indicated that alexithymia served as a significant mediator. Furthermore, the present findings also corroborate that deficits in attentional control constitute a significant risk factor for short-form video addiction. This aligns with prior research showing that individuals with weaker attentional control exhibit impaired cognitive regulatory function and struggle to inhibit dominant external responses. Short-form video applications, with their fragmented, fast-paced content and algorithmic personalization, provide a continuous stream of novel and high-intensity stimuli, which constitute potent distractions that individuals with limited attentional control find difficult to resist (Yan et al., 2024).

The second key finding of this study is that alexithymia served as a significant mediator in the relationship between attachment anxiety and short-form video addiction (AA → Ale → SVA). This result is strongly supported by existing literature. Numerous studies have demonstrated that individuals with higher levels of attachment anxiety tend to exhibit more severe alexithymic traits (Di Pentima and Toni, 2025). According to the self-medication hypothesis, addictive behaviors often function as maladaptive strategies for coping with negative emotions (Wang et al., 2025). Individuals with alexithymia lack effective emotion regulation skills and, when confronted with stress, are unable to manage discomfort through adaptive strategies such as introspection or emotional expression (Luminet and Nielson, 2025). In such contexts, short-form videos offer a highly appealing tool for emotional escape, allowing individuals with alexithymia to temporarily alleviate unbearable inner emptiness and discomfort, thereby achieving immediate emotional relief. Consequently, the present findings suggest that attachment anxiety may increase susceptibility to short-form video addiction by shaping individuals' emotional processing patterns, where deficient emotion regulation abilities lead them to rely on external “regulators” such as short-form videos to cope with negative affect.

Extending the two single mediation pathways, the primary finding of this study is that attachment anxiety is associated with short-form video addiction through a chain-mediated pathway involving attentional control and alexithymia (AA → AC → Ale → SVA). This pathway not only integrates the two previously identified parallel mediating mechanisms but also reveals a potential cascading relationship between cognitive deficits and emotional processing impairments. Although direct research on the impact of attentional control on alexithymia is limited, existing theoretical frameworks and indirect evidence provide a plausible rationale for this pathway.

One core feature of alexithymia is Externally Oriented Thinking (EOT), a cognitive style characterized by a focus on external details at the expense of internal emotional experience (Preece and Gross, 2024). The development of this thinking pattern may be associated with deficits in attentional control. Individuals with strong attentional control are able to flexibly shift focus between external stimuli and internal psychological states (Weinhardt et al., 2025). Conversely, individuals with poor attentional control are more readily “captured” by salient or novel external stimuli, making it difficult to voluntarily and consistently allocate attention toward introspection and reflection on internal emotional states (Luminet et al., 2024; Wang, 2025). Over time, this attentional pattern may solidify into an externally oriented cognitive habit, thereby exacerbating alexithymic traits.

Moreover, theoretical models such as the attention-appraisal model directly consider attentional deficits as a central component of alexithymia, suggesting that individuals with alexithymia struggle to focus attention on emotional information (Telli and Bilge, 2025). Therefore, the chain-mediated pathway identified in the present study suggests a novel mechanism: attachment anxiety first impairs domain-general cognitive control abilities (i.e., attentional control), which in turn hinders effective introspection, promoting domain-specific deficits in emotional cognition (i.e., alexithymia). Ultimately, this deficiency in emotion regulation drives individuals to seek external compensatory strategies, such as engaging with short-form videos, to modulate affective states.

Cultural context may further inform the interpretation of the present findings. In China, social norms often emphasize emotional moderation and interpersonal harmony, which can shape patterns of attentional control and emotional processing (Song et al., 2024). Such cultural tendencies may help explain why individuals with higher attachment anxiety in our sample tended to exhibit lower attentional control and higher alexithymia, which in turn were associated with greater engagement with short-form videos. Specifically, culturally influenced habits of managing internal distress through less direct emotional expression may contribute to reliance on external strategies, such as immersive digital media, for momentary emotional relief (Cho et al., 2025; Ford and Mauss, 2015; Ip et al., 2021). While culture is not a causal factor in these associations, acknowledging it provides a useful perspective for understanding the patterns observed and situates the findings within a broader socio-cultural context. Future research could further explore how culturally shaped norms interact with individual differences in attachment and self-regulation to influence short-form video engagement.

Several limitations of the present study should be acknowledged. First, the sample was gender-imbalanced, with a higher proportion of male participants, which may affect the generalizability of the findings. Second, all data were collected through self-report questionnaires, which are susceptible to social desirability and recall biases. Third, the cross-sectional design of the study does not allow for causal inferences regarding the associations among attachment anxiety, attentional control, alexithymia, and short-form video addiction. Future research could benefit from more balanced samples, multi-method assessments (e.g., behavioral tasks or peer reports), and longitudinal designs to further examine these relationships.

## Conclusion

In summary, this study systematically examined the associations among attachment anxiety, attentional control, alexithymia, and short-form video use by employing an integrated chain-mediated model. The main theoretical contribution of the present research lies in demonstrating that attachment anxiety, a distal factor rooted in early interpersonal experiences, is associated with greater engagement in short-form video use and with differences in self-regulatory capacities at both the cognitive (attentional control) and emotional (alexithymia) levels. These findings highlight the importance of considering mediating mechanisms when investigating the associations between distal factors, such as personality traits and attachment patterns, and outcomes like short-form video use. Ignoring these mediating variables may obscure the relationships between distal factors and outcome variables.

From a practical perspective, the present findings suggest potential targets for interventions. Given that attachment anxiety is associated with attentional control and alexithymia, interventions targeting individuals with higher levels of attachment anxiety could benefit from addressing these factors. For example, attentional control may be strengthened through cognitive training programs aimed at improving inhibitory and shifting capacities, while emotional processing abilities could be enhanced through psychological interventions such as mindfulness-based approaches or emotion-focused therapy, which foster skills in emotion identification, description, and regulation. This “dual-pathway” intervention strategy may be useful in addressing the associations between attachment anxiety and short-form video engagement.

## Data Availability

The raw data supporting the conclusions of this article will be made available by the authors, without undue reservation.
